# Seroprevalence of Leptospirosis among High-Risk Individuals in Morocco

**DOI:** 10.1155/2020/5236045

**Published:** 2020-05-20

**Authors:** Mohamed El Azhari, Mathieu Picardeau, Imad Cherkaoui, Mohamed Anouar Sadat, Houda Moumni, Kamal Marhoum El Filali, Hassan Ghazal, Abderrahmane Maaroufi, Salsabil Hamdi, Naima El Mdaghri, Pascal Bourhy

**Affiliations:** ^1^Laboratory of Bacteriology, Institut Pasteur Du Maroc, MoH, 1, Place Charles Nicolle, Casablanca 20360, Morocco; ^2^National Reference Center and The WHO Collaborating Center for Leptospirosis, Institut Pasteur, Paris, France; ^3^Directorate of Epidemiology and Disease Control, MoH, Casablanca, Morocco; ^4^Health Delegation of Mediouna, MoH, Casablanca, Morocco; ^5^Infectious Diseases Unit, Ibn Rochd University Hospital, Casablanca, Morocco; ^6^National Center for Scientific and Technological Research, Rabat, Morocco; ^7^Environmental Health Laboratory, Institut Pasteur Du Maroc, MoH, 1, Place Charles Nicolle, Casablanca 20360, Morocco

## Abstract

**Background:**

Leptospirosis is an anthropozoonotic reemerging neglected infectious disease underreported in most developing countries. A cross-sectional study was performed between 17 and 23 February 2014 to estimate the seroprevalence of leptospirosis among high-risk populations in Casablanca (Morocco).

**Methods:**

A total of 490 human serum samples (97.6% males) were collected in 3 high-risk occupational sites including the biggest meat slaughterhouse (*n* = 208), a poultry market (*n* = 121), and the fish market (*n* = 161). A total of 125 human blood samples were also collected from the general population and used in this study as a control group. To detect the presence of anti-*Leptospira*, sera were screened with in-house IgG and IgM enzyme-linked immunosorbent assay (ELISA). Positive samples were tested by Microscopic Agglutination Technique (MAT) using a panel of 24 serovar cultures and cut point of 1 : 25.

**Results:**

Seroprevalence of leptospirosis among the control group was 10.4% (13/125). A high seropositivity among the overall seroprevalence of 24.1% (118/490) was observed in the high-risk groups of which 7.3% (36/490), 13.7% (67/490), and 3.1% (15/490) were for anti-*Leptospira* IgM, IgG, and both IgG and IgM antibodies, respectively. Most of the positive individuals were occupationally involved in poultry (37.2%), followed by the market fish (26.1%) and the meat slaughterhouse (14.9%) workers. Among all ELISA-positive serum samples, 20.3% (*n* = 24) had positive MAT responses, of which the Icterohaemorrhagiae (*n* = 7) is the most common infecting serogroup followed by Javanica (4), Australis (2), and Sejroe, Mini, and Panama (one in each). In the remaining 8 MAT-positive sera, MAT showed equal titers against more than one serogroup.

**Conclusion:**

Individuals engaged in risk activities are often exposed to leptospiral infection. Therefore, control and prevention policies toward these populations are necessary.

## 1. Introduction

Leptospirosis, caused by pathogenic members of genus *Leptospira*, is the most widespread bacterial zoonosis [1]. Globally, this reemergence sickness is likely influenced by environmental conditions, such as occupation conditions, urbanization, and poverty [2, 3]. Lack of basic sanitation, poor housing conditions, and limited access to education and health increase the risk of human infection in urban areas [4, 5].

Its transmission in humans usually occurs through direct or indirect contact with the urine of an infected animal [6]. Although the infection may be prevalent in both urban and rural settings, it depends on animal contact. Environmental and socioeconomic conditions also facilitate infection. According to the World Health Organization, in a population, some high-risk groups are more likely to be exposed to *Leptospira* like veterinarians, fishermen, butchers, and poultry and animal farm workers [7]. The disease caused by a protean starts as an acute, generalized illness with symptoms usually confused with influenza in humans. However, it can progress to severe complications such as acute renal failure, pulmonary hemorrhage, and cardiac complications [8].

Annually, an estimated half a million cases of severe leptospirosis are reported globally [9]. However, despite its global importance, large gaps persist in the burden and epidemiological data about this disease in Africa [10]. In most African countries including Morocco, lack of data about this disease may be the consequence of lack of awareness of the disease among physicians, and nonspecific symptoms, limited access to diagnosis, and leptospirosis burden assessment studies.

In Morocco, since 1995, the notification system of mandatory notifiable humans infectious diseases including leptospirosis has been managed by the Direction of Epidemiology and Disease Control (Direction de l'Epidémiologie et de la Lutte contre les Maladies) [11]. The diagnosis of leptospirosis was based on a clinical picture of patients with an IgM-ELISA serology. To generate data about leptospirosis, routine data collected from clinical and laboratory structures during 2005 to 2012 period were analysed. A total of 305 human cases were reported during this period corresponding to an estimated annual incidence of 0.1 per 100 000. Similar results were previously/recently reported in temperate countries. In this Moroccan study, a seasonal increase of cases was observed during the summer-autumn period with regular epidemic peaks in some provinces with highest rate (17.0%). Most reported cases originating from the region of Casablanca were living in particular areas close to high-risk occupational sites like Sidi Moumen and Hay Mohammadi. In 2014, the Direction of Epidemiology and Disease Control and Institut Pasteur du Maroc in collaboration with the local authorities decided to carry out a seroepidemiological investigation on the suspected sites of Casablanca. The objective of this investigation was to estimate the seroprevalence of *Leptospira* spp. among various human high-risk groups and to determine the pathogenicity of *Leptospira* serovars. This study also aimed to generate information on risk factors related to this disease to promote public awareness since extensive professional activities are undertaken without adequate hygienic conditions.

## 2. Material and Methods

### 2.1. Study Site

In Casablanca, a cross-sectional study was carried out from 17 to 23 February 2014 to assess the seroprevalence of leptospirosis in 3 high-risk occupational sites (the biggest abattoir, the biggest poultry market, and the great harbour fish market) and in a general population not exposed to the risk of leptospirosis as control group.

### 2.2. Study Population

In this study, we enrolled adult participants from 3 occupational high-risk sites of leptospirosis and a control group. Recruitment of the participants was done after signing the written consent. The study design was submitted and approved by the Ethics Committee of Casablanca Medicine University of Morocco. The study was conducted in accordance with the principles of Helsinki Declaration. Participation to our study was voluntary, anonymous, confidential, and only for research purposes to protect privacy and ensure data integrity.

### 2.3. Sample Size and Power Calculation

In the absence of any data about estimates of the seroprevalence of leptospirosis in Morocco, we considered first a convenience global sample of about 500 individuals in the 3 high-risk occupational sites based on 80% power, a type I error of 5%, a 10% nonresponse rate, and an expected seroprevalence around 12% according to published international studies [12–14]. We have therefore expected our samples as follows: 250 subjects at the biggest abattoir, 125 subjects in the poultry market, and 125 subjects in the port, according to the number of employees in each site. A total of 125 subjects were also provided from the general population.

### 2.4. Data Collection

The questionnaire was designed by researchers. The interviews were conducted in Arabic. It includes information about work-related risk factors including work positions, occupational animal contact, personal protective equipment (e.g., gloves, etc.), and personal data such as age, gender, grade level, type and place of residence, and prior diagnosis of leptospirosis.

### 2.5. Serological Testing

5 ml blood was collected from each participant by venipuncture in a sterile tube and kept cool (5°C). After centrifugation, sera collected were kept frozen at −20°C until use. Serology for leptospirosis was performed at the National Reference Center for Leptospirosis at Institut Pasteur of Paris, France. Enzyme-linked immunosorbent assay (ELISA) and Microscopic Agglutination Technique (MAT) tests were carried out. IgM and IgG titers were determined separately by in-house ELISA, as previously described [15]. A subset of workers' sera that showed to contain antibodies to *Leptospira* spp. by IgM and/or IgG-ELISA were subsequently tested by MAT according to standard method [15], using 24 live leptospiral strains as antigens. A titer of 1 : 25 and higher was considered as evidence of *Leptospira* spp. infection as recommended for apparently healthy humans following the guidelines by the National Reference Laboratory for Leptospirosis of the Institut Pasteur in Paris. [16]. Positive samples were titrated up to the end titers. If more than 1 serogroup had the same high titer, the identification was labelled mixed reaction or indeterminate [17]. Patients whose sera were positive by IgM- and/or IgG-ELISA were considered as cases of leptospirosis.

### 2.6. Statistics

Data analysis was performed using Epi Info version 6 (Center for Disease Control and Prevention (CDC), Atlanta, USA). The results were stated as percentage, and the variables were measured using Chi-square (*χ*^2^), 95% confidence interval, and *P* value. The statistical significance was considered when the probability value was equal to or less than 0.05.

## 3. Results

The present work is a cross-sectional study that includes samples selected from 3 high-risk occupational sites among high-risk populations in Casablanca (Morocco) to estimate the seroprevalence of leptospirosis. A total of 490 workers participated in the study comprising 208 (42.5%) meat slaughterhouse workers, 121 (24.7%) poultry market workers, and 161 (32.8%) fish market workers and were investigated between 17 and 23 February 2014. Subjects were between 19 and 75 years old (mean age: 45.1 years). 478 (97.6%) workers were males compared to 12 (2.4%) females; the male to female ratio was 39.8 : 1. Because of the small number of female participants, no sex-stratified risk analysis for infection with *Leptospira* spp. was performed. Globally, the age group “40–60 years old” was predominant (58.2%), followed by the age group “19–39 years old” (34.5%) and the age group “61 years old and over” (7.3%). By occupational site, age group “40–60 years old” was found significantly predominant in slaughterhouse workers and in fish market workers Table 1. Most workers have only attained lower primary school and none of them had prior diagnosis of leptospirosis. They are mainly from Casablanca and are living in particular popular neighbourhoods as Lamdina lakdima, Hay Mohammadi, Moulay Rachid, Lalla Meryem, Lahraouiyine, and Sidi Moumen and close to high-risk occupational sites. The 125 subjects in the control group (general population) were between 21 and 68 years old (mean age: 42 years). 68 (54.4%) subjects were males compared to 57 (45.6%) females; the male to female ratio was 1.19 : 1. They were all living in the city of Casablanca, with no antecedent of leptospirosis and with a school level from primary to higher education.

Overall, 24.1% (*n* = 118) of workers scored positive for IgG and/or IgM anti-*Leptospira* antibodies, of which 7.3% (36/490) were positive for anti-*Leptospira* IgM antibodies, 13.7% (67/490) positive for IgG, and 3.1% (15/490) positive for both IgG and IgM. The poultry market workers had significantly the highest seropositivity (37.2%) as compared to that of fish market workers (26.1%) and meat slaughterhouse workers (14.9%) (*P* < 10^−4^); see Table 2. The distribution of positive samples sera by age and occupational groups is also shown in Table 2. With regard to the occupational groups, *Leptospira* IgM seroprevalence was 7.7% (*n* = 16) in meat slaughterhouse, 8.3% (*n* = 10) in poultry market, and 6.2% (*n* = 10) in fish market. *Leptospira* IgG seroprevalence was 5.3% (*n* = 30), 21.5% (*n* = 26), and 18.6% (*n* = 11) in fish market, poultry market, and meat slaughterhouse, respectively. Seroprevalence of both IgM and IgG was 1.9% (*n* = 4), 7.4% (*n* = 9), and 1.2% (*n* = 2) in meat slaughterhouse, poultry market, and at fish markets, respectively. Regarding the age groups in the three occupational groups, the highest seroprevalence of IgM and/or IgG antibodies was detected among workers whose age was between 40 and 60 years, followed by age group “19 to 39 years old.”

The distribution of ELISA titer among the 51 IgM positive workers' samples (36 IgM positive and 15 IgM/IgG positive sera) was as follows: 1 : 200 (74.5%), 1 : 400 (19.6%), 1 : 800 (3.9%), and 1 : 1600 (2.0%). Remarkably, the highest titer of IgM antibody was detected in only 3 (5.9%) samples sera (2 with titer of 1 : 800 and 1 with 1 : 1600) and the lowest titer (1 : 200 to 1 : 400) was found in the remaining 48 samples.

Of the 82 IgG positive workers samples (67 IgG positive and 15 IgM/IgG positive sera), the distribution of positivity scale titers was as follows: + was found in 41.5%, ++ in 29.3%, +++ in 14.6%, and ++++ in 11.0%. Most of the positive samples sera had low titers, with 34 (42.5%) and 24 (30.0%) samples having positivity scale titers of + and ++, respectively. IgG antibody titers were ranged on a scale from negative (0) to ++++ (0: negative, +: lightly positive, ++: averagely positive, +++: enough positive, and ++++: highly positive).

Among the 125 participants of the normal population, 6 and 7 male patients were, respectively, positive for IgM (age <40 years) and IgG (age 40–60 years). The titer of IgM antibodies varies between 1 : 200 (*n* = (4) and 1 : 400 (*n* = 2) while that of IgG varies between + (*n* = 4) and ++ (*n* = 3). The seroprevalence of 10.4% (13/125) in the control group was found significantly lower than the overall seroprevalence of 24.1% (118/490) in the high-risk groups.

All 118 ELISA-positive workers samples sera were tested by MAT and anti-leptospiral antibodies against one or more serovars were found in only 24 (20.3%) samples.  Table 3 shows the seroprevalence (MAT-positive) among different groups. It was significantly highest among the meat slaughterhouse workers (29.0%) followed by the poultry market workers (24.4%) and the fish market workers (9.5%) (*P*=0.01).

The MAT was not performed for the 13 IgM or IgG positive cases of the normal population.

Taking MAT titer of 1 : 25 as positive, the serogroup of *Leptospira* responsible for infection in the various occupational groups, as determined by the highest MAT titers, is summarized in Table 4.

The most common serogroup was Icterohaemorrhagiae, which was seen in 7 (29.1%), followed by Javanica in 4 (16.7%). Australis was seen in 2 (8.3%) and Sejroe (Hardo Prajitno), Mini, and Panama were seen in one each (4.2%). In the remaining 8 samples sera, MAT showed equal titer against more than one serovar (undetermined serogroup). That is, 5 seropositive sera had two agglutinations with Icterohaemorrhagiae serogroup: serogroups found in these 5 associations were Javanica (3 cases), Louisiana (3), Canicola (2), Mini (1), Batavia (1), and Javanica (1). In the remaining 3 undetermined serogroups, MAT showed equal titer against only two serogroups, Icterohaemorrhagiae with Canicola or Louisiana and Panama with Mini.

The distribution of MAT titer among these 24 samples is shown in Table 5. Only one has a titer of 1 : 200, three have titer of 1 : 100, and the remaining twenty had titer in the range of 1 : 25 to 1 : 50.

Remarkably, in the current study, 94 of all ELISA-positive samples sera could not be confirmed by the MAT. However, using ELISA, some of those sera were detected with significant titers of anti-*Leptospira* spp. antibodies, especially 6 MAT-negative IgM-ELISA-positive samples sera which were with titer equal to 400 (2 meat slaughterhouse workers sera, 3 poultry market workers sera, and 1 fish market worker serum) and 11 other MAT-negative IgG-ELISA-positive samples with elevated titer of IgG antibodies (1 meat slaughterhouse worker serum, 8 poultry market workers sera, and 2 fish market workers sera) (data not shown).

## 4. Discussion

Leptospirosis is one of the most common and widespread zoonotic infections in the world and is recognized as a neglected disease by the World Health Organization [18]. Any type of contact with animals poses the risk of acquiring leptospiral infection; in most instances it is acquired through environment contaminated with infected animal urine [6].

As far as we are aware, the current work is the first to assess the seroprevalence of *Leptospira* spp. in populations in Morocco. This seroprevalence is defined on the basis of ELISA outcomes. The reason for that quite simply is that such methodology is well suggested by some authors for determining serum antibody anti-*Leptospira* spp. [15, 19], more so as subjects of this investigation are apparently wholesome and Morocco is not an endemic area for this disease. Even more importantly, IgM and IgG antibodies remain for years in cases of leptospirosis [20, 21]. In addition, the detection of IgM antibody specific to *Leptospira* by ELISA has been widely used. There is no need to test a second sample if IgM-ELISA is positive, whereas paired sera testing is required for diagnostic confirmation by MAT assay [22]. Finally, it is known that the MAT, which detects both antibodies, lacks sensitivity and specificity when early acute-phase serum specimens alone are tested rather than paired specimens [8]. MAT sensitivity is also compromised if all locally relevant serovars are not represented [23].

The seroprevalence of 24.1% detected amongst high-risk participants is significantly higher than the seroprevalence in the control group and comparable to the 26.5% reported for the veterinary students in Trinidad and Tobago [24] and to 24% stated for healthy population in India [25], where IgG-ELISA and IgG/IgM-ELISA tests were performed, respectively. However, unlike Morocco, in India and Trinidad and Tobago, human leptospirosis is an endemic disease [25, 26].

One factor contributing to the rate of workers seropositivity in the present investigation could be the study population consisting chiefly of men (97.6%) with an average age of 45.1 years. Previous studies showed that the predominance of men among clinical case-patient is well recognized [21, 27, 28] and has been explained by their greater tendency to participate in high-risk outdoor exposure activities [27, 29]. Similarly, case rates among adults in the working-age population are also consistently the highest reported [21, 28]. Another potentially important factor contributing to this rate may be the educational status of most participants. Undoubtedly, the low educational attainment of these workers may be tantamount to lack of knowledge about avoidable possible risk factors associated with *Leptospira* infection.

Regarding the three occupational areas, the higher seropositivity rates detected among the poultry market workers and secondarily the fish market subjects compared to the meat slaughterhouse participants indicate that work in the first two sites is a significant risk factor for leptospiral infection. These two workplaces which lack hygienic conditions are suitable for the survival of a large number of rodents and provide them an abundant source of food, as well as for the dogs, cats, and other urban reservoirs, such as horses and donkeys, which are commonly encountered in the same places of work. As a consequence, these bad working conditions may represent a proper environment for the survival of leptospires and allow both areas to be an epidemiological niche for frequent transmission of those bacteria. Workers which are constantly exposed to this harsh environment are always within poor hygienic conditions and almost none of them used any kind of protective measure while working. All the more so, some of them were with injury, wound, or cut at the hands, which again increases the chances of entry of the *Leptospira* spp. [27, 30, 31]. To all of this, the repeated contact with the same circulating spirochetes leads the immune system to regularly produce anti-*Leptospira* antibodies and attenuating the symptoms in case of a reinfection [21]. Much more, the first infection may have acted as a natural live vaccine conferring cross protection among unrelated *Leptospira* serovars [21]. Accordingly, subjects who have participated in this study can be asymptomatic for leptospirosis, despite the fact that they were positive in serology.

We recorded a much lower prevalence in meat slaughterhouse when compared to the two previous areas. These discrepancies in prevalence rates are likely to be related to the hygienic conditions. The great slaughterhouse in Casablanca is versatile, with modern materials and transparent slaughter circuits which meet the sanitary hygiene. However, the finding rate is higher when compared to that reported in Colombia (7%) for the group of people working in slaughterhouses [32].

On the other hand, the result of this work showed that Icterohaemorrhagiae is the first major infecting serogroup. This MAT result is in agreement with many other reports from different parts of the world [21, 24, 33, 34]. One study conducted in Morocco, approximately 32 years ago, revealed that such serogroup was most frequently diagnosed and was responsible for a severe disease: 1 out of 3 patients was hospitalized in intensive care unit [35]. This datum illustrates that at least in the late 1970s and early 1980s, Icterohaemorrhagiae was the main serogroup responsible for human leptospirosis in Morocco.

However, a few unexpected observations from our study were the wide differences in seroprevalence rates between ELISA and MAT tests. Of the 118 positive ELISA, 94 samples workers sera were MAT-negative. A logical reasoning could be that other serovars or serogroups, not tested for in the current study, were responsible for the remaining ELISA-positive MAT-negative samples. That assumption might be argued by the data released by Bakoss et al., these authors have reported that the number and types of serovars causing leptospirosis in different geographical locations affect reported seroprevalence by the MAT [36]. The second reasonable explanation for this wide discrepancy is the high sensitivity and low specificity of ELISA compared to MAT [24]. Thirdly, the numerous MAT undetermined serogroup with titers 1 : 25 (*n* = 8) might indicate that these patients were previously infected by other serovars. It was reported in one recent study that the newly acquired serovar may have cross-reaction with the former infecting serovar [22]. In this case, titer of antibody specific to previous serovar could be higher than of antibody against the new infecting serovar. Four-fold rising antibody titer will be used as an indicator of current infection and all serovars that provide four-fold rising antibody titer or higher should be considered [22]. Finally, it may mean that the seropositive workers with low level of antibodies (IgG and/or IgM) had a condition other than leptospirosis or that their immune system is not responding normally.

The diagnostic procedure used in the current study may be considered a limitation because it tested for IgM and IgG in a single sample rather than paired samples from each patient. Thus, the results cannot be generalized to the overall Moroccan population. We plan to perform a panel that cover the Morocco Leptospira strains to identify the circulating serogroups and also to find out whether the MAT panel covered the all serogroups.

## 5. Conclusion

The present results indicated that leptospirosis is a potential health hazard of occupational groups in Casablanca. The prevention of occupational human exposure is recognized as a basic control measure.

The present results indicated that leptospirosis is a potential health concern of occupational groups in Casablanca. Individuals engaged in risk activities are often exposed to this infectious occupational zoonotic disease. Therefore, policy of basic control and prevention toward these populations are necessary and largely recommended to keep workers and others healthy and safe. Health authorities must enhance workers awareness of this disease, its means of transmission and the precautions to be taken through sensitization about the good working practices and guidelines to follow.

## Figures and Tables

**Table 1:** Demographic characteristics of workers participating in the leptospirosis study in Casablanca (Morocco) (*N* = 490).

Variables	Slaughterhouse workers (*N* = 208) *n* (%)	Poultry market workers (*N* = 121) *n* (%)	Fish market workers (*N* = 161) *n* (%)	Total (%) (*N* = 490) *n* (%)	*P* value^*∗*^
Sex					N A
Male	205 (98.6)	116 (95.9)	157 (97.5)	478 (97.6)	
Female	3 (1.4)	5 (4.1)	4 (2.5)	12 (2.4)	

Age (years)					10^−5^(^*∗*^)
19–39	57 (27.4)	58 (47.9)	54 (33.6)	169 (34.5)	
40–60	146 (70.2)	52 (43.0)	87 (54.0)	285 (58.2)	
>60	5 (2.4)	11 (9.1)	20 (12.4)	36 (7.3)	

^*∗*^Statistically highly significant (*P* < <<0.05), NA : not Applicable.

**Table 2:** Leptospiral seropositivity rates according to antibodies (IgM/IgG), age groups and occupational risk in Casablanca (Morocco).

Antibodies	Age group (years)	Control group (*N* = 125) *n* (%)	Slaughterhouse workers (*N* = 208) *n* (%)	Poultry market workers (*N* = 121) *n* (%)	Fish market workers (*N* = 161) *n* (%)	Total workers (*N* = 490) *n* (%)
IgM (-) IgG (-)	19 ≥ 60	112 (89.6)	177 (85.1)	76 (62.8)	119 (73.9)	372 (75.9)
IgM (+) IgG (-)	19–39	6 (4.8)	6 (2.9)	4 (3.3)	4 (2.5)	14 (2.9)
	40–60	0 (0.0)	10 (4.8)	4 (3.3)	5 (3.1)	19 (3.9)
	>60	0 (0.0)	0 (0.0)	2 (1.7)	1 (0.6)	3 (0.6)
Sub total		6 (4.8)	16 (7.7)	10 (8.3)	10 (6.2)	36 (7.3)
IgM (-) IgG (+)	19–39	0 (0.0)	2 (1.0)	8 (6.6)	7 (4.3)	17 (3.5)
	40–60	7 (5.6)	8 (3.8)	17 (14.0)	18 (11.2)	43 (8.8)
	>60	0 (0.0)	1 (0.5)	1 (0.8)	5 (3.1)	7 (1.4)
Sub total		7 (5.6)	11 (5.3)	26 (21.5)	30 (18.6)	67 (13.7)
IgM (+) IgG (+)	19–39	0 (0.0)	1 (0.5)	4 (3.3)	0 (0.0)	5 (1.0)
	40–60	0 (0.0)	3 (1.4)	5 (4.1)	2 (1.2)	10 (2.0)
	>60	0 (0.0)	0 (0.0)	0 (0.0)	0 (0.0)	0 (0.0)
Sub total		0 (0.0)	4 (1.9)	9 (7.4)	2 (1.2)	15 (3.1)
All seropositivity^*∗*^	19 ≥ 60	13 (10.04)	31 (14.9)	45 (37.2)	42 (26.1)	118 (24.1)

^*∗*^Chi^2^ = 11.12, *P* < 10^−3^.

**Table 3:** Seroprevalence (MAT positivity) of leptospirosis among different occupational groups in Casablanca (Morocco).

Occupational groups	No. tested	Positive (%)	95 confidence interval (%)
Meat slaughterhouses workers	31	9 (29.0)	14.88–48.23
Poultry market workers	45	11 (24.4)	13.38–39.86
Fish market workers	42	4 (9.5)	3.09–23.55
Total	118	24 (20.3)	13.71–28.95

Chi^2^ = 0.54, *P*=0.01.

**Table 4:** Distribution of different serogroups of leptospira among humans seropositive occupational groups of Casablanca (Morocco).

Serogroups	High-risk group			Total (%)
	Poultry market workers (%)	Fish market workers (%)	Meat slaughterhouse workers (%)	
Icterohaemorrhagiae	5 (45.5)	1 (25.0)	1 (11.1)	7 (29.1)
Javanica	1 (9.1)	1 (25.0)	2 (22.2)	4 (16.7)
Australis	2 (18.2)	0 (0.0)	0 (0.0)	2 (8.3)
Sejroe (hardo prajitno)	0 (0.0)	1 (25.0)	0 (0.0)	1 (4.2)
Mini	1 (9.1)	0 (0.0)	0 (0.0)	1 (4.2)
Panama	0 (0.0)	0 (0.0)	1 (11.1)	1 (4.2)
Undetermined serogroup	2 (18.2)	1 (25.0)	5 (55.6)	8 (33.3)
Total (%)	11 (100.0)	4 (100.0)	9 (100.0)	24 (100.0)

**Table 5:** Distribution of leptospiral serovars in the various occupational groups with the highest titers as determined by MAT.

Study site	No	Age	Gender	IgM-ELISA	IgG-ELISA	MAT	
				Titer	Positivity scale^(a)^	Serogroup	Titer
Meat slaughterhouse	68	51	M	<200	+	Icterohemorrhagiae	50
	78	56	M	<200	+	US^(b)^	25
	95	37	M	200	0	Javanica	25
	101	58	M	200	+	US	25
	112	45	M	400	++	US	25
	116	43	M	200	0	US	25
	123	25	M	400	0	Panama	25
	131	45	M	400	0	Javanica	25
	171	53	M	400	0	US	25

Poultry market	210	33	M	<200	+	Icterohemorrhagiae	100
	239	38	M	200	0	Australis	25
	260	55	M	200	+++	Australis	50
	261	27	M	800	++++	Icterohemorrhagiae	200
	264	25	M	<200	+	US	25
	274	31	M	200	++	Mini	50
	279	54	M	<200	++	Icterohemorrhagiae	50
	282	37	M	<200	+	Icterohemorrhagiae	100
	291	57	M	1600	+++	Javanica	25
	301	63	M	<200	+++	US	25
	324	60	M	800	++++	Icterohemorrhagiae	100

Fish market	338	35	M	<200	++	Icterohemorrhagiae	50
	415	73	M	<200	+	US	25
	425	39	M	<200	++	Sejroe (hardo prajitno)	50
	441	53	M	200	++	Javanica	25

^(a)^ 0: negative, +: lightly positive, ++: averagely positive, +++: enough positive, ++++: highly positive. ^(b)^ US: undetermined serogroup.

## Data Availability

The datasets analysed during the current study are available from the corresponding author on reasonable request.
